# The Effects of Zinc-Containing Mouthwashes on the Force Degradation of Orthodontic Elastomeric Chains: An *In Vitro* Study

**DOI:** 10.1155/2022/3557317

**Published:** 2022-04-29

**Authors:** Ali R. Issa, Ammar S. Kadhum, Shahbaa A. Mohammed

**Affiliations:** Department of Orthodontics, College of Dentistry, University of Baghdad, Baghdad, Iraq

## Abstract

**Objective:**

This study aimed to evaluate and compare the force degradation of two types of elastomeric chains following different periods of immersion in zinc-containing mouthwashes.

**Materials and Methods:**

Four hundred and forty pieces of Elasto-Force and Super Elasto-Force elastomeric chains were divided into two control and eight experimental groups. The pieces were stretched to 25 mm on pins mounted on an acrylic block and stored in distilled water at 37°C. The experimental groups were immersed in four different types of mouthwash for one minute twice a day throughout the test period. Ten continuous thermocycles per day between cold and hot water baths (5–55°C) were carried out. Forces were measured at six-time intervals (initial, 24 hours, 1, 3, 6, and 8 weeks). The mean force was calculated and compared among different elastomeric chains, mouthwashes, and times using the *t*-test and one-way ANOVA test followed by Tukey's HSD test. The level of significance was set at 0.05.

**Results:**

Both types of elastomeric chains had significant force degradation over time (74–79% at 8 weeks). The Super Elasto-Force generated a higher force level than the Elasto-Force elastomeric chain at all time points. SmartMouth Clinical DDS mouthwash had a significantly lower effect on force degradation than other mouthwashes with no significant difference compared to control groups.

**Conclusions:**

Depending on these results: there is no clinically significant difference between both types of elastomeric chains, although Super Elasto-Force delivered a higher force level. The pH of the mouthwashes could play a role in force degradation over time, rather than other ingredients including zinc. The SmartMouth mouthwash had the minimum effect on force degradation of elastomeric chains, followed by Halita, Listerine Total Care Zero, and Breath Rx, respectively.

## 1. Introduction

Elastomeric chains had been introduced into orthodontic treatment since 1960s [1] and were used for different purposes, including generalized space closure, correcting midline, closure of extraction space, and moving impacted teeth [2]. They are widely used since they are reasonably hygienic, cost-effective, simple to use, and do not require patient cooperation [1].

Elastomeric chains are made of polyurethane and lose their force over time because of their viscoelastic properties [3]. Several factors can affect this force degradation as temperature [4], pH changes [5], and free radicals [6]. Mouthwashes used by patients during orthodontic therapy could result in force degradation due to any of the aforementioned factors. Orthodontists usually recommend the use of fluoridated mouthwash to aid in the prevention of white spots and dental caries after the placement of fixed orthodontic appliances [7]. As well as using chlorhexidine mouthwash for a short-term (usually 14 days) for noncompliant patients with persistent plaque accumulation is recommended [8]. In addition to white spot lesions and dental caries, fixed orthodontic treatment is a risk factor for oral malodor [9]. It is well-known that some metal ions, particularly zinc (Zn), reduce or inhibit the forming of volatile sulphur compounds (VSCs) [10, 11], just like some antibacterial agents such as chlorhexidine and cetylpyridinium chloride (CPC), with a subsequent decrease in oral malodor [11].

Many studies investigated the effect of fluoridated [12–15], alcohol-containing [3, 16], and chlorhexidine mouthwashes [12, 17] with contradicting results between significantly affecting force degradation or no effect.

Javanmardi and Salehi [13] studied the effect of zinc-containing mouthwash on the elastomeric chain, and they found lower force degradation than other experimental and even control groups. They proposed that the presence of zinc could be the cause of their findings and suggested further studies. As there is limited information about the effect of zinc-containing mouthwashes on the elastomeric chains, this study was carried out to evaluate and compare the effect of four different zinc-containing types of mouthwashes on the force degradation of two types of elastomeric chains.

## 2. Materials and Methods

In this study, two short transparent elastomeric chains with different designs were used; Elasto-force (EF) and Super Elasto-Force (SEF) (Dentaurum, Ispringen, Germany). Four different types of mouthwash were used namely: SmartMouth Clinical DDS (with cetylpyridinium chloride (CPC) and zinc ion), Halita® (chlorhexidine-based), Breath Rx (CPC), and Zytex (a blend of zinc, and thymol and eucalyptus essential oils), and Listerine Total Care Zero® (fluoride-containing mouthwash).

A total of 440 pieces were used and divided equally between EF and SEF. Each type of elastomeric chain had 5 groups; a control group where the elastomeric chain was immersed in distilled water (DW) and four test groups, one for each mouthwash type. Each piece of elastomeric chain was five loops long, with an extra half loop at each end to prevent the probable damage during handling [12].

The pH of each mouthwash and the distilled water was measured using a digital pH meter (GOnDO, PL-700PC, Taipei/Taiwan) and is shown in Table 1.

Ten acrylic blocks with 20 stainless-steel pins were used to hold the elastomeric pieces stretched in place. The 20 pins were arranged in two parallel rows 25 mm apart, representing the distance between canine and first molar teeth [18]. All of the 440 pieces were mounted on the pins in the same manner, and their residual forces were measured at six different time intervals (time 0 in dry condition, 1 day, 1 week, 3 weeks, 6 weeks, and 8 weeks) using a digital force gauge (Weiheng, China).

During force measurement, the acrylic block was securely bound to a benchtop using a holding clamp. The tensile force was measured by attaching one end of the elastomeric chain to the force tester while the other end is held in position by the pin. The force measurement was done on twenty pieces (ten from each type) at the initial (in dry condition) and another 20 pieces at 1 day (only stored in distilled water) time points. Then, at each time point, 100 pieces were measured for residual force as the exposure to mouth washes begin just after 1 day time point force measurement.

Throughout the test duration, all samples of elastomeric chains (except those tested for initial force) were stored in plastic containers filled with distilled water and incubated at 37°C. To simulate oral temperature changes, after the force measurement at day-1, all samples were subjected to 10 continuous thermocycles per day between cold (5°C) and hot (55°C) water baths with 30 seconds dwell time in every single bath and exchange time of 30 seconds [12, 19].

The experimental samples were removed from distilled water and immersed in the corresponding mouthwash solution for 1 minute every 12 hours; this procedure was repeated daily from day-2 till the end of the study, and the mouthwashes were replenished between intervals. After immersion, they were transported to another distilled water container, specific for each mouthwash for 30 minutes to simulate the use of the mouthwash by the patient [13]. At the end, after rinsing with water, they were returned to the main container and incubated at 37°C. The control samples followed the same procedure as experimental samples, but instead of mouthwash, they were exposed to distilled water.

### 2.1. Statistical Analysis

The statistical package for social sciences (SPSS Inc., version 26, Chicago, Illinois, USA) was used to carry out the statistical procedures. The data distribution was assessed using the Shapiro–Wilk test. An independent sample *t*-test was used to statistically compare the means of force level of the two types of elastomeric chains. One-way analysis of variance (ANOVA) test was used to assess the statistical significance of the difference in the mean of force level of elastomeric chains at different time intervals and different chemical solutions. Then, Tukey's HSD test post hoc analysis was used when there was a significant difference in the ANOVA test. The level of significance was set at 0.05.

## 3. Results

**Figure 1:**
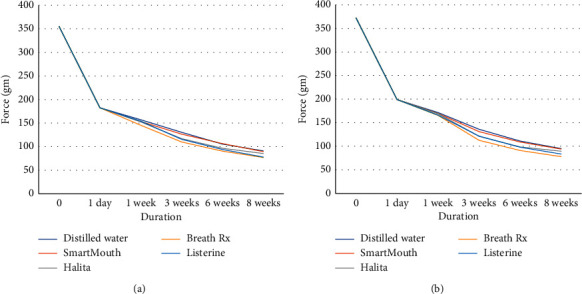
Mean values of the force delivered from Elasto-Force (a) and Super Elasto-Force (b) elastomeric chains treated with different chemical solutions.

Shapiro–Wilk test was conducted and revealed that the data were normality distributed. The mean, standard deviation (SD), and percentage of force degradation of the force values for two types of the short transparent elastomeric chains immersed in each chemical solution at various time intervals are presented in Table 2. Effects of time were statistically significant at all time points and all groups (Figure 1 and Tables 3 and 4).

An independent sample *t*-test was used for comparison between elastomeric chain types and is summarized in Table 5.

The effect of different chemical solutions was statistically significant for both types of elastomeric chains and at all time points (Table 6). At one-week time point, Tukey's HSD test shows no significant differences in the mean force of both types of elastomeric chains among the tested solutions except between Breath Rx and distilled water for both types of elastomeric chains, and Breath Rx with SmartMouth, Halita, and Listerine total care zero for Elasto-Force type. At 3, 6, and 8 weeks time points, with exception of SmartMouth mouthwash, all other tested mouthwashes show significant differences in the mean force of elastomeric chains as compared to distilled water (Table 7).

## 4. Discussion

This study was carried out in an *in vitro* setting in an attempt to minimize the effects of other confounding variables like pH, wet condition, stretching, temperature, and mastication. The elastomeric chains were tested at six-time points (initial, 1 day, 1 week, 3 weeks, 6 weeks, and 8 weeks) to monitor the changes that occur over time between adjustment appointments while also comparing the results obtained by other studies [20–25]. The 6 and 8 week time points were also chosen as an endpoint according to Proffit et al. [8], who considered it a more typical appointment cycle.

All of the tested mouthwashes are available over-the-counter, and the manufacturer places no restrictions on their usage period, unlike chlorhexidine mouthwashes (0.2 and 0.12% concentrations), which are advised not to be used for more than two consecutive weeks to avoid their adverse effects [26].

The initial force values ranged from 355 ± 4.1 gm to 372 ± 4.8 gm; which means both types of chains delivered force levels above what is considered clinically acceptable for the movement of a group of teeth or a single tooth (300 gm), as suggested by Quinn and Yoshikawa [20], and Lotzof et al. [21]. After 24 hours Elasto-Force elastomeric chains were performing at approximately 182.5 gm (51.41% residual force) and Super Elasto-Force elastomeric chains at 199 gm (53.49% residual force), which is considered to be physiologically acceptable [20, 21]. These observed reductions in force at the first 24 hours are in the range of 50–80% residual force values reported by Baty et al. [22]. The average residual force of the elastomeric chains was 32–37% after three weeks of activation. Grassi et al. [23] reported a reduction in the range of 20–40%, Aldrees et al. [24] reported a reduction of 45%, and De Genova et al. [4] reported a slightly larger range from 39.1 to 56%. Oshagh and Ajami [25], on the other hand, found a substantially lower percentage of remaining force by the end of the third week of activation at 15.84%. Baty et al. [22] considered 100 gm as the lower limit of force level for physiologically acceptable tooth movement. At 6 weeks time point, only control groups and the chains treated with SmartMouth mouthwash showed a mean load equal to or above this value, and after 8 weeks, all groups ended below this recommended force level. Two significant mechanisms may account for this force degradation pattern of the elastomeric chain over time: the stretching mechanism of the elastomeric chain and absorption of the fluid. Stretching a chain stresses the molecular polymer within it, leading to chain slippage, sliding of polymer molecules that passed one another, broken primary bonds, and the appearance of permanent deformation [27]. In addition to the stretching mechanism, the second factor that affects the force degradation pattern is the absorption of the fluid, which has a plasticizer effect [28].

Differences in the magnitude of delivered force were noted among two types of elastomeric chains. The amount of force delivered by the Super Elasto-Force elastomeric chain type was higher than the Elasto-Force one at all time points (Table 2). Although the distance between the lumen of the loops of the chains is equal, the size of the loops and links are larger in Super Elasto-Force elastomeric chains than in Elasto-Force elastomeric chains, which seems to be the cause of this difference. The result of this study is contrary to Aldrees et al. [24] study. They studied the color stability and force degradation of 19 types of clear elastomeric chains for 4 weeks. According to their study, the Super Elasto-Force chain showed lower mean load and force degradation than the Elasto-Force type at all time points [24]. These differences could be attributed to methodological variances, various initial forces, environmental circumstances, and different force measurement apparatus.

In the present study, the results showed that both chain types treated with mouthwashes presented lower force levels than chains exposed to distilled water only. However, the groups treated with SmartMouth showed no statistically significant differences with control groups in all time intervals, contrary to all the other experimental groups (Table 7).

Previously, the effect of sodium fluoride and chlorhexidine mouthwashes on force degradation of elastomeric chains was evaluated by other investigators. However, a debate about the results of these studies is present. Omidkhoda et al. [12] and Menon et al. [15] reported a significant force degradation after immersion of chains in sodium fluoride mouthwashes, while Javanmardi and Salehi [13], and Mirhashemi et al. [14] found no effect. Behnaz et al. [7] studied the effect of whitening (Listerine® Healthy White) and daily sodium fluoride (Listerine® Total Care Zero) mouthwashes on the force degradation of elastomeric chains. They concluded that the daily use of Listerine® Total Care Zero mouthwash could increase force degradation of elastomeric chains, which is consistent with the finding of the present study. Although, they did not consider its pH or the other ingredients contained within the mouthwash.

Pithon et al. [17] studied the effect of 4 types of chlorhexidine with different concentrations and formulations on elastomeric chains. They found that all tested mouthwashes showed no significant effect on force degradation of elastomeric chains. Their finding is in agreement with Mirhashemi et al. [14]. In contrast, Omidkhoda et al. [12] in a study conducted in 2015, reported a significant effect of chlorhexidine which could be attributed to ethanol content (13.65%) of the studied mouthwash; as the effect of alcohol on the force degradation of the elastomeric chains was reported by Larrabee et al. [3] and Mahajan et al. [16]; it seems that the significant difference seen between Halita mouthwash groups and control groups of the present study might not be due to the presence of chlorhexidine in this mouthwash, especially as its concentration is low 0.05%.

The pH of mouthwashes could be one of the influencing factors, as Pureprasert et al. [29] reported that exposure to sodium hydroxide (NaOH), an alkaline solution, lowered the maximum forces and delivery forces of various elastic bands. Despite that, Lacerda dos Santos et al. [30] found that this effect was not significant when observed at weak acidic and neutral pH (5.0, 6.0, and 7.5 pH). However, Clemitson [31] reported that polyurethane material can easily be hydrolyzed when exposed to an acidic pH below <5.4 or pH above >8.0. In accordance with this and the pH level of the mouthwashes used in this study (Table 1), it might indicate that the acidic condition of these mouthwashes was giving more negative effects than Zinc ion or other ingredients.

More research is needed to ascertain the effect of the tested mouthwashes on elastomeric chain force degradation *in vivo*. Until proven otherwise, the current practice of replacing elastomeric chains every 3 weeks when using either Halita, Listerine Total Care Zero, or Breath Rx and every 6 weeks when SmartMouth mouthwashes are prescribed to the patient, seems logical.

## 5. Conclusions

1. The highest rate of force degradation occurred during the first 24 hours, in a range of 46%–49%, followed by a steadier and more gradual rate over the entire testing period.
2. Although Super Elasto-Force chain produced a higher force level than the regular one at all time points, no clinically significant difference between both types of elastomeric chains was present; as both types are capable to maintain the force level between 100 and 300 gm for 6 weeks, which is clinically accepted to provide adequate force for orthodontic tooth movement.
3. The pH of the mouthwashes could play a role in force degradation over time rather than other ingredients including zinc, chlorhexidine, fluoride, essential oils, and cetylpyridinium chloride; so, whenever possible, the pH of the mouthwashes should be taken into consideration during their prescription.
4. The SmartMouth mouthwash had the least effect on force degradation of elastomeric chains, followed by Halita, Listerine Total Care Zero, and Breath Rx, respectively.

## Figures and Tables

**Table 1:** pH of each chemical solution.

Chemical	pH
Distilled water	7.0
SmartMouth	5.45
Halita	5.25
Breath Rx	4.75
Listerine	4.48

**Table 2:** Mean, SD, and percentage of force degradation of the force generated by Elasto-Force and Super Elasto-Force elastomeric chains treated with different chemical solutions.

Duration	Elastic type	Distilled water		SmartMouth		Halita		Breath Rx		Listerine	
		Mean ± SD (gm)	%^*∗*^	Mean ± SD (gm)	%	Mean ± SD (gm)	%	Mean ± SD (gm)	%	Mean ± SD (gm)	%
Initial	EF	355 ± 4.08	—	355 ± 4.08	—	355 ± 4.08	—	355 ± 4.08	—	355 ± 4.08	—
	SEF	372 ± 4.83	—	372 ± 4.83	—	372 ± 4.83	—	372 ± 4.83	—	372 ± 4.83	—
1 day	EF	182.5 ± 5.4	48.59	182.5 ± 5.4	48.59	182.5 ± 5.4	48.59	182.5 ± 5.4	48.59	182.5 ± 5.4	48.59
	SEF	199 ± 5.16	46.5	199 ± 5.16	46.5	199 ± 5.16	46.5	199 ± 5.16	46.5	199 ± 5.16	46.5
1 week	EF	157 ± 4.21	55.77	153.5 ± 4.74	56.76	152.5 ± 4.24	57.04	145.5 ± 4.97	59.01	154 ± 4.59	56.62
	SEF	171.5 ± 4.74	53.9	169.5 ± 4.37	54.43	169 ± 3.94	54.57	165.5 ± 5.98	55.51	166 ± 3.94	55.38
3 weeks	EF	130.5 ± 3.68	63.24	126.5 ± 3.37	64.34	117 ± 4.21	66.98	110 ± 3.33	69.01	115.5 ± 3.68	67.46
	SEF	136 ± 3.94	63.44	131 ± 4.59	64.78	121 ± 3.94	67.47	112.5 ± 3.53	69.76	121.5 ± 5.29	67.34
6 weeks	EF	105.5 ± 3.68	70.28	106.5 ± 4.11	70	96.5 ± 4.74	72.82	90.5 ± 3.68	74.51	94 ± 3.94	73.52
	SEF	111 ± 5.16	70.16	109 ± 4.59	70.7	98.5 ± 5.79	73.52	91 ± 4.59	75.54	98 ± 3.49	73.66
8 weeks	EF	91 ± 3.94	74.37	89.5 ± 3.68	74.79	85.5 ± 3.68	75.91	77 ± 3.49	78.31	78 ± 4.21	78.03
	SEF	95 ± 4.08	74.46	94 ± 3.16	74.73	89.5 ± 4.37	75.94	78 ± 4.21	79.03	83 ± 4.83	77.69

%^∗^ indicates the percentage of force degradation; SD: standard deviation; EF: Elasto-Force; SEF: Super Elasto-force.

**Table 3:** Effect of time intervals on the force level of two types of elastomeric chains.

Media	Elastic type	ANOVA test	
		*F* test	*p* value
Distilled water	Elasto-Force	773.603	≤0.001^*∗*^
	Super Elasto-Force	847.221	≤0.001^*∗*^
SmartMouth	Elasto-Force	734.332	≤0.001^*∗*^
	Super Elasto-Force	957.065	≤0.001^*∗*^
Halita	Elasto-Force	801.564	≤0.001^*∗*^
	Super Elasto-Force	1000.438	≤0.001^*∗*^
Breath Rx	Elasto-Force	1014.174	≤0.001^*∗*^
	Super Elasto-Force	1157.750	≤0.001^*∗*^
Listerine	Elasto-Force	952.462	≤0.001^*∗*^
	Super Elasto-Force	1098.408	≤0.001^*∗*^

^*∗*^Significance at *p* < 0.05.

**Table 4:** Tukey's HSD test for comparison between each two-time interval.

Time 1	Time 2	Tukey's HSD test									
		Distilled water		SmartMouth		Halita		Breath Rx		Listerine	
		EF	SEF	EF	SEF	EF	SEF	EF	SEF	EF	SEF
1 day	1 week	≤0.001^*∗*^	≤0.001^*∗*^	≤0.001^*∗*^	≤0.001^*∗*^	≤0.001^*∗*^	≤0.001^*∗*^	≤0.001^*∗*^	≤0.001^*∗*^	≤0.001^*∗*^	≤0.001^*∗*^
	3 weeks	≤0.001^*∗*^	≤0.001^*∗*^	≤0.001^*∗*^	≤0.001^*∗*^	≤0.001^*∗*^	≤0.001^*∗*^	≤0.001^*∗*^	≤0.001^*∗*^	≤0.001^*∗*^	≤0.001^*∗*^
	6 weeks	≤0.001^*∗*^	≤0.001^*∗*^	≤0.001^*∗*^	≤0.001^*∗*^	≤0.001^*∗*^	≤0.001^*∗*^	≤0.001^*∗*^	≤0.001^*∗*^	≤0.001^*∗*^	≤0.001^*∗*^
	8 weeks	≤0.001^*∗*^	≤0.001^*∗*^	≤0.001^*∗*^	≤0.001^*∗*^	≤0.001^*∗*^	≤0.001^*∗*^	≤0.001^*∗*^	≤0.001^*∗*^	≤0.001^*∗*^	≤0.001^*∗*^

1 weeks	3 weeks	≤0.001^*∗*^	≤0.001^*∗*^	≤0.001^*∗*^	≤0.001^*∗*^	≤0.001^*∗*^	≤0.001^*∗*^	≤0.001^*∗*^	≤0.001^*∗*^	≤0.001^*∗*^	≤0.001^*∗*^
	6 weeks	≤0.001^*∗*^	≤0.001^*∗*^	≤0.001^*∗*^	≤0.001^*∗*^	≤0.001^*∗*^	≤0.001^*∗*^	≤0.001^*∗*^	≤0.001^*∗*^	≤0.001^*∗*^	≤0.001^*∗*^
	8 weeks	≤0.001^*∗*^	≤0.001^*∗*^	≤0.001^*∗*^	≤0.001^*∗*^	≤0.001^*∗*^	≤0.001^*∗*^	≤0.001^*∗*^	≤0.001^*∗*^	≤0.001^*∗*^	≤0.001^*∗*^

3 weeks	6 weeks	≤0.001^*∗*^	≤0.001^*∗*^	≤0.001^*∗*^	≤0.001^*∗*^	≤0.001^*∗*^	≤0.001^*∗*^	≤0.001^*∗*^	≤0.001^*∗*^	≤0.001^*∗*^	≤0.001^*∗*^
	8 weeks	≤0.001^*∗*^	≤0.001^*∗*^	≤0.001^*∗*^	≤0.001^*∗*^	≤0.001^*∗*^	≤0.001^*∗*^	≤0.001^*∗*^	≤0.001^*∗*^	≤0.001^*∗*^	≤0.001^*∗*^

6 weeks	8 weeks	≤0.001^*∗*^	≤0.001^*∗*^	≤0.001^*∗*^	≤0.001^*∗*^	≤0.001^*∗*^	≤0.001^*∗*^	≤0.001^*∗*^	≤0.001^*∗*^	≤0.001^*∗*^	≤0.001^*∗*^

^*∗*^Significance at *p* < 0.05; EF: Elasto-Force; SEF: Super Elasto-force.

**Table 5:** Descriptive statistics and comparison of the force level between Elasto-Force and Super Elasto-Force elastomeric chains.

Media	Time	EF	SEF	Comparison		
		Mean ± SD (gm)	Mean ± SD (gm)	Mean difference (gm)	*t*-test	*p* value
Distilled water	1 day	182.5 ± 5.40	199 ± 5.16	−16.5	−6.983	≤0.001^*∗*^
	1 week	157 ± 4.21	171 ± 4.74	−14	−7.225	≤0.001^*∗*^
	3 Weeks	130.5 ± 3.69	136 ± 3.94	−5.5	−3.220	0.005^*∗*^
	6 Weeks	105.5 ± 3.69	111 ± 5.16	−4.5	0.22	0.013^*∗*^
	8 Weeks	91 ± 3.94	95 ± 4.08	−4	−0.194	0.039^*∗*^

SmartMouth	1 day	182.5 ± 5.4	199 ± 5.16	−16.5	−6.983	≤0.001^*∗*^
	1 week	153.5 ± 4.74	169.5 ± 4.38	−16	−7.838	≤0.001^*∗*^
	3 Weeks	126.5 ± 3.37	131 ± 4.6	−4.5	−2.496	0.022^*∗*^
	6 Weeks	106.5 ± 4.12	109 ± 4.6	−2.5	−1.282	0.216
	8 Weeks	89.5 ± 3.69	94 ± 3.16	−4.5	−2.929	0.009^*∗*^

Halita	1 day	182.5 ± 5.4	199 ± 5.16	−16.5	−6.983	≤0.001^*∗*^
	1 week	152.5 ± 4.25	169 ± 3.94	−16.5	−9.000	≤0.001^*∗*^
	3 Weeks	117 ± 4.22	121 ± 3.94	−4	−2.191	0.042^*∗*^
	6 Weeks	96.5 ± 4.74	98.5 ± 5.8	−2	−0.844	0.410
	8 Weeks	85.5 ± 3.67	89.5 ± 4.38	−4	−2.209	0.040^*∗*^

Breath Rx	1 day	182.5 ± 5.4	199 ± 5.16	−16.5	−6.983	≤0.001^*∗*^
	1 week	145.5 ± 4.97	165.5 ± 5.99	−20	−8.127	≤0.001^*∗*^
	3 Weeks	110 ± 3.33	112 ± 3.53	−2.5	−1.627	0.121
	6 Weeks	90.5 ± 3.69	91 ± 4.59	−0.5	−0.268	0.791
	8 Weeks	77 ± 3.5	78 ± 4.22	−1	−0.577	0.571

Listerine Total Care Zero	1 day	182.5 ± 5.4	199 ± 5.16	−16.5	−6.983	≤0.001^*∗*^
	1 week	154 ± 4.59	166 ± 3.94	−12	−6.267	≤0.001^*∗*^
	3 Weeks	115.5 ± 3.69	121.5 ± 5.3	−6	−2.939	0.009^*∗*^
	6 Weeks	94 ± 3.94	98 ± 3.5	−4	−2.400	0.027^*∗*^
	8 Weeks	78 ± 4.22	83 ± 4.83	−5	−2.466	0.024^*∗*^

^*∗*^Significance at *p* < 0.05; SD: standard deviation; EF: Elasto-Force; SEF: Super Elasto-force.

**Table 6:** Effect of different chemical solutions on Elasto-Force and Super Elasto-Force elastomeric chains.

Time	Elastic type	ANOVA test	
		*F* test	*p* value
1 week	EF	8.701	≤0.001^*∗*^
	SEF	13.696	0.032^*∗*^
3 weeks	EF	46.168	≤0.001^*∗*^
	SEF	45.79	≤0.001^*∗*^
6 weeks	EF	30.581	≤0.001^*∗*^
	SEF	30.29	≤0.001^*∗*^
8 weeks	EF	46.535	≤0.001^*∗*^
	SEF	45.428	≤0.001^*∗*^

^*∗*^Significance at *p* < 0.05; EF: Elasto-Force; SEF: Super Elasto-force.

**Table 7:** Tukey's HSD test for comparison between each two solutions.

Media		1 week		3 weeks		6 weeks		8 weeks	
		EF	SEF	EF	SEF	EF	SEF	EF	SEF
Distilled water	SmartMouth	0.436	0.872	0.125	0.088	0.981	0.882	0.903	0.983
	Halita	0.197	0.752	≤0.001^*∗*^	≤0.001^*∗*^	≤0.001^*∗*^	≤0.001^*∗*^	0.019^*∗*^	0.038^*∗*^
	Breath Rx	≤0.001^*∗*^	0.046^*∗*^	≤0.001^*∗*^	≤0.001^*∗*^	≤0.001^*∗*^	≤0.001^*∗*^	≤0.001^*∗*^	≤0.001^*∗*^
	Listerine	0.587	0.080	≤0.001^*∗*^	≤0.001^*∗*^	≤0.001^*∗*^	≤0.001^*∗*^	≤0.001^*∗*^	≤0.001^*∗*^

SmartMouth	Halita	0.988	0.999	≤0.001^*∗*^	≤0.001^*∗*^	≤0.001^*∗*^	≤0.001^*∗*^	0.150	0.130
	Breath Rx	0.003^*∗*^	0.322	≤0.001^*∗*^	≤0.001^*∗*^	≤0.001^*∗*^	≤0.001^*∗*^	≤0.001^*∗*^	≤0.001^*∗*^
	Listerine	0.999	0.457	≤0.001^*∗*^	≤0.001^*∗*^	≤0.001^*∗*^	≤0.001^*∗*^	≤0.001^*∗*^	≤0.001^*∗*^

Halita	Breath Rx	0.011^*∗*^	0.457	≤0.001^*∗*^	≤0.001^*∗*^	0.015^*∗*^	0.009^*∗*^	≤0.001^*∗*^	≤0.001^*∗*^
	Listerine	0.947	0.606	0.891	0.999	0.664	0.999	≤0.001^*∗*^	0.009^*∗*^
Breath Rx	Listerine	≤0.001^*∗*^	0.999	0.014^*∗*^	≤0.001^*∗*^	0.317	0.017^*∗*^	0.353	0.073

^*∗*^Significance at *p* < 0.05; SD: standard deviation; EF: Elasto-Force; SEF: Super Elasto-force.

## Data Availability

The data that support the findings of this study are available from the corresponding author upon request.
